# Standardized MRI nomenclature for knee meniscal lesions: SSR consensus panel report

**DOI:** 10.1007/s00256-026-05281-5

**Published:** 2026-06-12

**Authors:** Jie C. Nguyen, Tetyana Gorbachova, Dyan V. Flores, Bethany U. Casagranda, Majid Chalian, Kimia K. Kani, Megan K. Mills, Ogonna Kenechi Nwawka, Kevin G. Shea, Kurt P. Spindler, Kathryn J. Stevens, Jennifer S. Weaver, Adam C. Zoga, Robert D. Boutin

**Affiliations:** 1 Department of Radiology, Children’s Hospital of Philadelphia, Philadelphia, PA USA; 2 Perelman School of Medicine, University of Pennsylvania, Philadelphia, PA USA; 3 Present Address: Department of Radiology, Jefferson Einstein Hospital, Jefferson Health, Sidney Kimmel Medical College at Thomas Jefferson University, Philadelphia, PA USA; 4 Department of Radiology, University of Ottawa, Ottawa, Canada; 5 Department of Radiology, Allegheny Health Network, Pittsburgh, PA USA; 6 Department of Radiology, University of Washington, Seattle, WA USA; 7 Department of Diagnostic Radiology and Nuclear Medicine, University of Maryland School of Medicine, Baltimore, MD USA; 8 Department of Radiology, University of Utah, Salt Lake City, UT USA; 9 Department of Radiology and Imaging, Hospital for Special Surgery, New York, NY USA; 10 Department of Orthopaedic Surgery, Stanford University School of Medicine, Stanford, CA USA; 11 Department of Orthopaedic Surgery, The Cleveland Clinic, Cleveland, OH USA; 12 Department of Radiology, Stanford University School of Medicine, Stanford, CA USA; 13 Department of Radiology, University of Texas at San Antonio, San Antonio, TX USA; 14 Department of Radiology, Thomas Jefferson University Hospital, Sidney Kimmel Medical College at Jefferson, Philadelphia, PA USA

**Keywords:** Consensus, Knee, Meniscus, MRI, Nomenclature

## Abstract

**Objective:**

To assess nomenclature variability and develop recommendations on standardized MRI reporting of meniscal findings.

**Methods:**

The Society of Skeletal Radiology identified standardized MRI reporting of knee menisci as an important topic for study and invited all members to serve on a panel to provide consensus recommendations. The Society empaneled 12 musculoskeletal radiologists and 2 orthopaedic surgeons. The panel reviewed published literature (PubMed, Scopus, and Embase) using predetermined criteria for inclusion (peer-reviewed, English-language, human studies) and exclusion (case reports, conference abstracts, book chapters, expert opinions, and commentaries). Literature analysis focused on nomenclature relevant to MRI reporting in two general domains: (I) meniscal anatomy and anatomic variants and (II) meniscal tears and associated findings.

**Results:**

Substantial nomenclature variability was identified across both domains. For anatomy and variants, inconsistencies involved root zone definitions, vascular zone descriptors, perimeniscal stabilizer terminology (including eponyms), and diagnostic criteria for the discoid meniscus. For tears and associated findings, variability involved numerical grading systems, the terms “complete” and “incomplete,” extrusion measurement methodology, and interpretive labels (e.g., “traumatic,” “degenerative,” “repairable,” “stable”). Ten consensus recommendations for standardized MRI reporting were developed.

**Conclusion:**

Descriptive anatomic language—specifying what is seen and where—can be more reproducible and clinically actionable than reporting variable numerical grades and evolving classifications. Adoption of our ten pragmatic recommendations has the potential to reduce miscommunication, improve inter-institutional consistency, and support clinical research.

## Introduction

The knee meniscus has key roles in maintaining joint health, including absorbing and distributing loads, contributing to joint congruity and stability, and promoting joint proprioception and nutrition. The meniscus has been highly conserved during evolution, with similar anatomy and function since human-like bipedal locomotion evolved approximately 2 million years ago [1].

Unlike the meniscus itself, the *terminology* to describe the meniscus has evolved tremendously. For example, the most common way to describe the “medial and lateral meniscus” for many centuries (from the 1540 s [2] through at least the 1940 s [3, 4]) was as the “internal and external semilunar cartilage.” In recent decades, meniscal terminology has evolved further and classification systems have proliferated in the medical literature. An unintended consequence of such “evolutionary drift” in medical language is inconsistent or imprecise communication, resulting in an unmet need to standardize terminology. Furthermore, while we recognize that classification systems can be particularly helpful in clinical trials, anatomically precise reporting is paramount. Many miscommunications can be avoided by simply describing what we see and where.

When interpreting a knee MRI, a standardized lexicon is fundamental to reporting both anatomic findings and clinically actionable derangements. To assess the current status of terminology standardization, our objectives were to evaluate nomenclature variability in the literature and to develop recommendations for standardized reporting of meniscal findings on knee MRI. Our panel summarizes relevant peer-reviewed literature and offers recommendations in two general areas: (I) meniscal anatomy and anatomic variants and (II) meniscal tears and associated MRI findings.

## Materials and methods

### Consensus paper panel and process

The Society of Skeletal Radiology (SSR) Standards and Guidelines Committee identified standardized MRI reporting of knee menisci as an important topic for study and invited all SSR members to serve on a consensus paper panel. A panel of 12 musculoskeletal radiologists and 2 orthopaedic surgeons with clinical and research expertise in the evaluation of meniscal derangements was selected by the Committee and tasked with developing the current interdisciplinary consensus document supported by peer-reviewed literature.

Members of the Panel communicated via electronic mail, videoconferencing, and in person from March 2023 to May 2026 to review relevant peer-reviewed literature for nomenclature variability and best practices for standardized MRI reporting of meniscal findings. Panelists also leveraged their clinical experiences with reporting variability and deliberated on opportunities for improving nomenclature standardization.

### Literature search

Literature searching was performed initially in June 2023 using PubMed, Scopus, and Embase databases using search terms that included combinations of the following root words, “knee,” “knee joint,” “menisc*,” “meniscus injury,” “meniscus lesion,” “meniscus trauma,” “tear,” “retear,” “composition,” “intrasubstance,” “myxoid,” “mucoid,” “degeneration,” “magnetic resonance*,” “magnetic resonance arthrogra*,” and “accuracy” to identify peer-reviewed publications. Predetermined criteria were used for publication inclusion (i.e., studies on humans and published in English-language) and exclusion (i.e., case reports, conference abstracts, book chapters, expert opinions, commentaries, unavailable full text). No patient age or publication date cutoffs were applied. Supplemental database searches were performed again in April 2026.

### Literature analysis

Literature analysis focused on nomenclature relevant to MRI reporting was subdivided into two domains: (I) meniscal anatomy and anatomic variants and (II) meniscal tears and associated findings. For each of these domains, subgroups of panel members were tasked with summarizing the published literature, identifying inconsistencies in terminology relevant to MRI reporting of meniscal lesions, presenting the existing evidence for Panel deliberation, and compiling a preliminary draft of recommended consensus statements. After modifications and review of recent Delphi-based interdisciplinary report [5], the final consensus report with recommendation statements was reviewed and approved by the Panel.

### Overview

Diagnostic and therapeutic techniques relating to the knee meniscus have accelerated rapidly in recent decades—and this trend is expected to continue. One of the driving forces behind accelerated medical understanding has been easy access to the world’s biomedical literature via databases like PubMed. PubMed, freely available online to the public since 1996, is currently a conduit to > 40 million biomedical citations, with more citations now added at a rate of ~2 million per year. For the keywords “knee” and “meniscus,” the upsurge in English language publications has been considerable, with 911 publications during 2025 (up from 3 in 1963, 149 in 1993, and 811 in 2023). With such abundant and evolving literature in mind, this report is not comprehensive and assumes a working knowledge of the meniscus on MRI.

While some themes in the literature have not substantially changed in recent years, our report highlights topics that are dynamic and clinically impactful. Recent publications show that there is surging interest in imaging and treatment of root and ramp injuries [6], meniscus extrusion [7], partial meniscectomy [8], meniscal tear repair [9], and meniscal centralization [10].

Consensus on consistent terminology usage has had a demonstrable, salutary impact on the practice of medicine [11]. Our report focuses on current nomenclature variability in the literature and recommendations for standardized MRI reporting of meniscal lesions. Table 1 summarizes a “top 10” list of important nomenclature issues in the MRI literature on the meniscus with reporting recommendations. Additional information can be found in recent authoritative reviews with standard nomenclature that summarize anatomy and injuries for posteromedial meniscocapsular ramp lesions [12], the medial meniscus (MM) [13], the lateral meniscus (LM) [14], and the discoid LM [15].

**Table 1:** “Top 10” list of important nomenclature issues in the meniscus MRI literature with reporting recommendations

*Meniscal roots*	For MRI findings at or near a root, it may be appropriate to describe the anatomic location of the finding relative to the tibial insertion (e.g., at the root attachment, at the root-posterior horn junction, or in the posterior horn near the root). For practical purposes, the MM posterior root zone currently is understood to include both the enthesis and the adjacent meniscus within 1 cm
*Meniscal vascularity*	For MRI reporting, the anatomic location of a meniscal finding can be described as involving the meniscocapsular junction, outer (peripheral) third, middle third, or inner third. We also recommend that radiologists remain conversant in the red/white terminology that endures as a prevailing nomenclature in clinical orthopaedics, MRI reporting, and related research
*Meniscal stabilizers*	Rather than eponyms and the historical term “coronary ligament”, the SSR Panel endorses transitioning to the anatomically appropriate terms “anterior meniscofemoral ligament”, “posterior meniscofemoral ligament”, and “meniscotibial ligament”
*Meniscal stabilizers*	Standardized nomenclature for the inferior and superior PMFs is recommended for future publications. For current clinical MRI reporting, however, description of PMF by location relative to the popliteal hiatus using any of the published synonyms remains acceptable
*Discoid meniscus*	Given that MRI protocols now generally use thinner (3–4 mm) slices, the “3 bowties” criterion for diagnosing a discoid LM should be abandoned. Currently, diagnosis of a discoid meniscus is appropriate on a mid-coronal slice when the width of the body segment measures 15 mm or more
*Intrameniscal signal alteration & numerical grading*	Clinical MRI reports should phase out numerical grading of meniscal signal in favor of descriptive text. Clear descriptive text emphasizing both surfacing signal and morphologic distortion criteria is recommended
*Tear classification systems*	Rather than rigidly adhering to a single classification system, we recommend using standardized descriptive terminology (e.g., horizontal, longitudinal-vertical, radial, complex) that draws from existing classification systems to provide a shared vocabulary relevant to clinical decision-making
*Tear patterns*	When reporting a meniscus tear, the descriptors “complete”, “incomplete”, or “partial” can be clarified by also specifying the tear pattern and extent
*Meniscal extrusion*	Extrusion should be reported using standardized terminology that includes the numeric measurement in millimeters, the compartment (medial or lateral), and the coronal reference slice (preferably at the mid-tibial plateau level), rather than relying on variable binary thresholds that differ across the literature
*Restraint in reporting*	MRI reports should describe meniscal tear morphology, location, and associated findings without inferring etiology ("traumatic" vs. "degenerative"), chronicity ("acute" vs. "chronic"), or surgical suitability ("repairable," "unrepairable," "stable," "unstable"). For the postoperative meniscus, the term "residual tear" should be avoided in favor of descriptive language that distinguishes expected postoperative change from findings suspicious for recurrent tear

Our results and recommendations are presented here for two general domains: (I) anatomy and anatomic variants and (II) meniscal tears and associated findings.

## Panel review and recommendations

### Anatomy and anatomic variants

In this section, we discuss anatomy related to meniscal roots, vascularity, stabilizers, and the most important congenital variant, the discoid LM.

Although historically divided into three segments, we recommend that each meniscus be subdivided into five major segments (from anterior to posterior): anterior root, anterior horn, body, posterior horn, and posterior root. Of note, there is substantial variability in the literature when referring to the body segment (e.g., “mid body,” “mid-body,” “midbody,” “midpart,” “pars intermedia”). No distinct boundaries separate the meniscal segments on MRI.

The fibrocartilaginous meniscus transitions from uncalcified to calcified fibrocartilage at a calcification tidemark (visible at microscopy); the calcified fibrocartilage then connects with the underlying cortical bone at the enthesis [16]. The biomechanical strength of this transitional zone is lower than the adjacent meniscus and its tibial attachment, making it more vulnerable to tearing [17].

#### Meniscal Roots

Broad consensus regarding the profound biomechanical importance of the roots has emerged over the past several decades, with clinical and imaging research showing root integrity is essential for converting compressive knee loading to normal circumferential (hoop) tensile stresses within the meniscus [18–22].

**Fig. 1:**
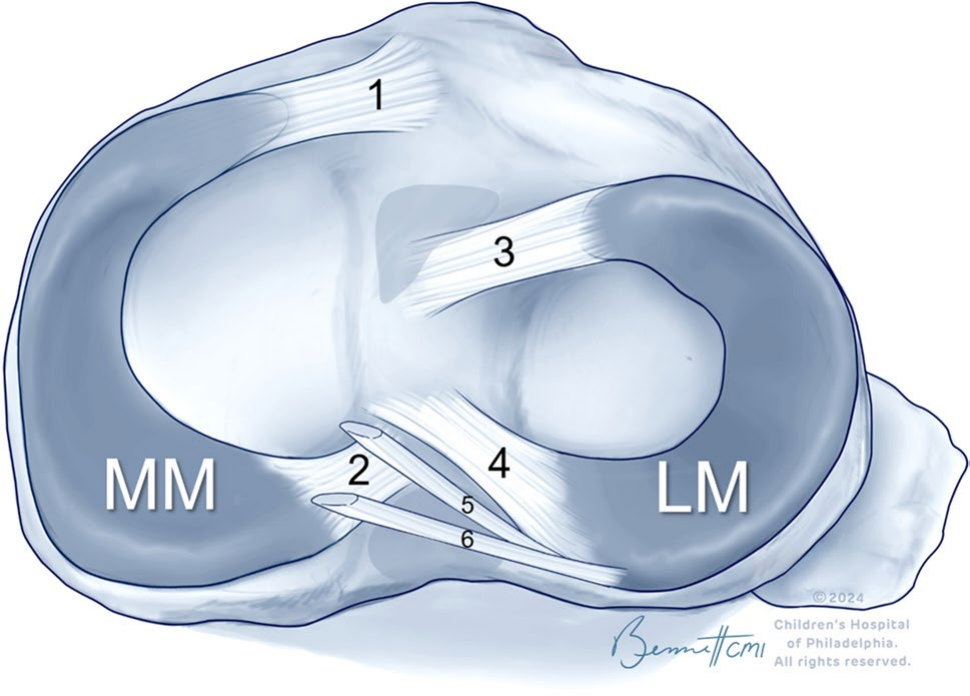
Illustration shows the anatomic footprints of the four roots inserting into the tibia: (1) Anterior root of MM. (2) Posterior root of MM. (3) Anterior root of LM. (4) Posterior root of LM. (5) Anterior meniscofemoral ligament (Lig. of Humphry). (6) Posterior meniscofemoral ligament (Lig. of Wrisberg). Illustration by B. Bennett, CMI @2024 The Children’s Hospital of Philadelphia. All rights reserved. Used with permission

The anatomic footprints for the four roots inserting into the tibia follow a consistent order from anterior to posterior: MM anterior root, LM anterior root, LM posterior root, and MM posterior root (Fig. 1). These entheses have consistent relationships relative to the tibial eminences and cruciate ligaments. For example, the LM anterior root attaches in the anterior intercondylar region at the posterolateral aspect of the ACL insertion and the MM posterior root attaches between the medial tibial eminence and the PCL [23–25].

On MRI, anatomic variations in the appearance of the root attachments to the tibia should be recognized as relatively common.

- Rather than appearing as uniformly hypointense, the root attachments normally can have a striated or “comblike” appearance owing to a multifascicular architecture. This MRI appearance is most common at the LM anterior root and should not be confused with a tear [6].
- Anatomic variations at the anterior roots are well known [24]. The MM anterior root often inserts near the anterior aspect of the medial tibial eminence, but also can insert more anteriorly, on the downsloping portion of the anterior tibial edge, in knees without meniscal tears (7% [26]–72% [23]). Such anterior positioning should not be confused with pathologic extrusion [27].
- The LM posterior root attachment onto the tibia at the intercondylar region is variable. Fascicular attachments ranging from one (24%–53%) to two (42%–76%) to three (2%–5%) are considered normal on MRI [23, 25, 28]. Multiple insertions may confer additional stability but also create complexity in diagnosing partial root tears. LM posterior root tears occur in 12%–15% of patients undergoing ACL reconstruction [14].

**Fig. 2:**
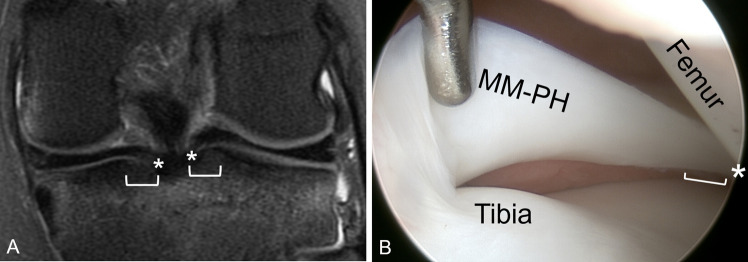
Coronal proton-density-weighted fat-suppressed MRI from a 22-year-old man (**A**) with ACL tear and arthroscopic view from a 12-year-old boy (**B**) show a normal posterior root of the medial meniscus which includes not only the entheseal attachment (*), but also the adjacent meniscus within 1 cm (brackets), continuous with the medial meniscus posterior horn (MM-PH). The entheseal attachment is a difficult region for the arthroscopist to visualize directly. Arthroscopic view courtesy of Theodore J. Ganley, MD

Inconsistent anatomic definitions of the meniscal root are present in the literature. Two general competing definitions of the meniscal root exist: an “anatomic definition” strictly limited to the tibial anatomic insertion, and a broader “arthroscopic definition” that includes up to 1 cm of adjacent meniscal tissue to capture an extended root zone regarded as relevant to biomechanics and patient management (Fig. 2). The “within 1 cm” definition of the root zone has been applied to all four roots [29].

Recent literature, however, suggests that the biomechanical significance of a meniscal tear depends more on radial width and orientation than on distance from the bony insertion per se, which challenges the rationale for defining the roots as extending a fixed distance of up to 1 cm into the meniscal tissue [30, 31]. The concept of a fixed “1 cm root zone” may ultimately be replaced by a more nuanced framework based on tear radial width, tear orientation, and the integrity of the perimeniscal stabilizers. At present, there is no single, anatomically grounded consensus [32], but the “within 1 cm” definition of the MM posterior root is most prevalent in the literature [33].

##### Recommendation #1

**Fig. 3:**
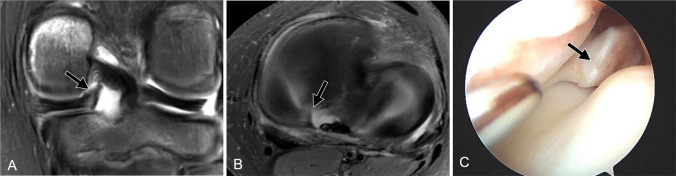
Medial meniscus posterior root avulsion in a 10-year-old boy. Coronal proton-density-weighted fat-suppressed MRI (**A**), axial proton-density-weighted fat- suppressed MRI (**B**), and arthroscopic view (**C**) demonstrate complete avulsion of the posterior root of the medial meniscus (arrows) at the enthesis

For MRI findings at or near a root, it may be appropriate to describe the anatomic location of the finding relative to the tibial insertion (e.g., at the root attachment, at the root-posterior horn junction, or in the posterior horn near the root) (Fig. 3). For practical purposes, the MM posterior root zone currently is understood to include both the enthesis and the adjacent meniscus within 1 cm.

### Meniscal Vascularity

**Fig. 4:**
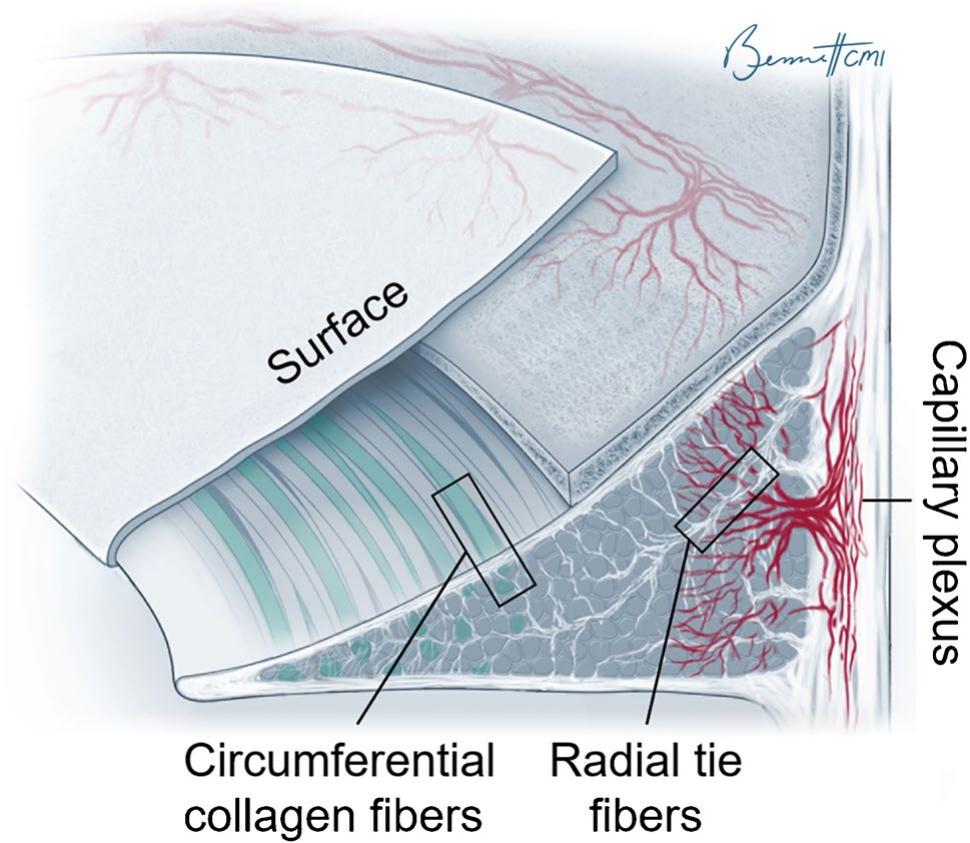
Histologic structure of the meniscus. Illustration of the cross-sectional anatomy of the meniscus depicting meniscal vascularity and organization of collagen fibers. Illustration by B. Bennett, CMI @2024 The Children’s Hospital of Philadelphia. All rights reserved. Used with permission

The vascular supply in the meniscus plays a key role in determining healing potential, including after surgical repair. The essential gradient is from the vascularized periphery to the avascular free edge (Fig. 4). The meniscus is commonly divided into circumferential zones based on vascularity in adults: the perimeniscal tissue at the meniscocapsular junction (zone 0, vascularized), the peripheral third (zone 1, “red-red zone,” vascularized), the middle third (zone 2, “red-white zone,” variable vascularity), and the inner third (zone 3, “white-white zone,” avascular) [34].

Peripheral vascularity, along with radial tie fibers [35], can account for the horizontally oriented increased signal intensity within the menisci that is commonly observed in zone 1 of the posterior horn near the meniscocapsular junction. This appearance is distinguished from a tear by its lack of extension to the articular surface. Most studies have emphasized that vascularity in zone 2 is negligible in adults [36], with the most cited study indicating that only the peripheral 10%–25% (i.e., < one-third) of the meniscus is vascularized [37]. However, studies since 2021 have emphasized that microvascularity can vary substantially [38–41], with decreased vascularization and cellular density generally associated with aging. 

Given this variability and that microvascularity is not visible on routine MRI [42], the International Society of Arthroscopy, Knee Surgery and Orthopaedic Sports Medicine (ISAKOS, Geneva, Switzerland) discourages “red” and “white” as descriptors. Rather, anatomic location along the radial axis of the meniscus can be reported unambiguously as the outer (peripheral) third, middle third, or inner third (near the free edge). We recognize, however, that many orthopaedists and radiologists find legacy terms convenient, such as “red-white zone junction” when reporting the “middle third” (as “middle third” could potentially be misunderstood as the body segment in the traditional three-segment framework that divides the meniscus into anterior horn, body, and posterior horn).

#### Recommendation #2

**Fig. 5:**
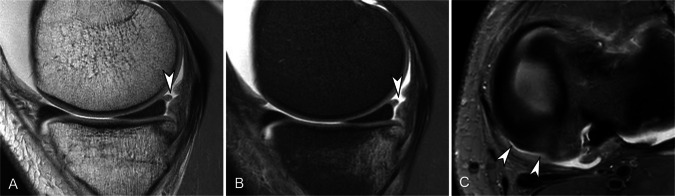
Meniscocapsular junction injury in a 25-year-old man who sustained ACL rupture while playing basketball. Sagittal proton-density-weighted MRI (**A**), sagittal T2-weighted fat-suppressed MRI (**B**), and axial proton-density-weighted fat-suppressed MRI (**C**) demonstrate full thickness longitudinal-vertical tear at the meniscocapsular junction of the posterior horn of the medial meniscus (arrowheads)

For MRI reporting, the anatomic location of a meniscal finding can be described as involving the meniscocapsular junction, outer (peripheral) third, middle third, or inner third (Fig. 5). We also recommend that radiologists remain conversant in the red/white terminology that endures as a prevailing nomenclature in clinical orthopaedics, MRI reporting, and related research [43–47].

#### Meniscal stabilizers

Meniscal stabilizers, including numerous perimeniscal attachments, can play an important role in meniscal functioning. Nomenclature inconsistencies identified in the literature generally reflect ongoing use of legacy terms or current areas of debate related to conflicting scientific evidence. Small anatomic structures may be challenging to study, and there are legitimate anatomic controversies and variations that are active areas of research [48, 49].

**Fig. 6:**
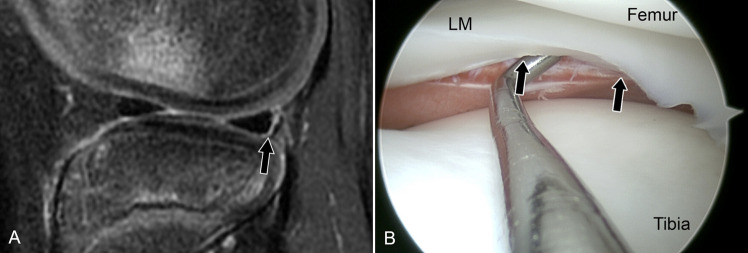
Meniscocapsular junction injury in a 12-year-old boy with an ACL tear. Sagittal proton-density-weighted fat-suppressed MRI (**A**) and arthroscopic view (**B**) show a longitudinal-vertical tear (arrows) of the posterior horn of the lateral meniscus (LM) near the meniscocapsular junction, with meniscal periphery contiguous with posterior meniscofemoral ligament. Arthroscopic view courtesy of Theodore J. Ganley, MD

The anterior and posterior meniscofemoral ligaments of the LM (often referred to as the ligament of Humphry and Wrisberg, respectively) pass anterior and posterior to the PCL. These structures should not be confused with an anterior horn meniscofemoral ligament (e.g., anteromedial meniscofemoral ligament), which is an uncommon structure with a distribution similar to an infrapatellar plica [50, 51]. The international standard is now to use descriptive anatomical nomenclature [23] rather than eponyms that often misattribute priority [52–54]. By extension, the colloquial “Wrisberg rip” can be replaced with anatomic language that describes a tear along the posterior meniscofemoral attachment to the LM (Fig. 6). As an alternative, patterns of injury in this region can be evoked by the “zip sign” [55] and “meniscus on a string” appearance [6].

**Fig. 7:**
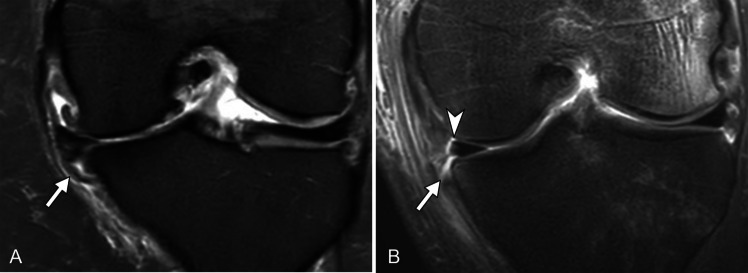
Meniscotibial ligament. Coronal proton-density-weighted fat-suppressed MRI of a 59-year-old woman (**A**) with complete tear of the posterior root of the medial meniscus and meniscal extrusion depicts medial meniscotibial ligament (arrow). Coronal proton-density-weighted fat-suppressed MRI of a 32-year-old man (**B**) with ACL tear demonstrates a tear of the medial meniscotibial ligament (arrow); note associated meniscocapsular tear (arrowhead)

The meniscotibial ligament (sometimes referred to as the coronary ligament) has been featured prominently in recent literature for its potential role in numerous derangements (e.g., meniscus extrusion, root tears, ramp lesions, osteoarthritis) [48, 56, 57] (Fig. 7). The status of the meniscotibial ligament may merit reporting in the setting of meniscal extrusion [58].

Meniscotibial ligament attachments vary substantially around the rim of the tibia. The deep MCL consists of two divisions: a meniscofemoral ligament superiorly and a meniscotibial ligament inferiorly. At the MM body region, the meniscotibial ligament attaches to the inferior margin of the meniscus and inserts approximately 4 mm distal to the tibial articular surface [56, 59]. At the MM posterior horn, the inferior and superior meniscus attachments are appropriately termed the “posterior meniscotibial ligament” and the “meniscocapsular junction,” respectively. Laterally, there is also a meniscotibial ligament, but it is notably absent near the popliteal hiatus at the posterolateral aspect of the knee.

##### Recommendation #3

Rather than eponyms and the historical term “coronary ligament,” the SSR Panel endorses transitioning to the anatomically appropriate terms “anterior meniscofemoral ligament,” “posterior meniscofemoral ligament,” and “meniscotibial ligament.”

**Fig. 8:**
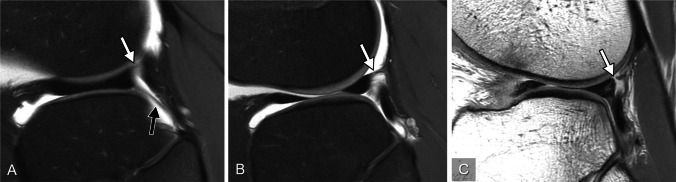
Popliteomeniscal fascicles. Sagittal T2-weighted fat-suppressed MR arthrographic images in a 15-year-old girl (**A**, **B**) show normal inferior (black arrow) and superior (white arrow) popliteomeniscal fascicles. Sagittal proton-density-weighted MRI of a 20-year-old man (**C**) with an acute ACL tear shows a torn superior popliteomeniscal fascicle (arrow); note associated anterior tibial translation owing to ACL deficiency

At the posterolateral corner, stabilizers of the LM have variable descriptions and evolving nomenclature [49, 60–65]. For example, fibrous connective tissue bands between the popliteus and LM posterior horn are now best referred to as popliteomeniscal *fascicles* (PMFs) (rather than *ligaments*), because they do not attach to bone. Two PMFs of the LM have been validated anatomically in numerous studies, with original description of fascicles at the floor and roof of the popliteal hiatus as “inferior” and “superior” fascicles, respectively (Fig. 8) [60]. Numerous subsequent descriptors have been used for the PMF “floor” (e.g., inferior, anteroinferior, anterior) and the PMF “roof” (e.g., superior, posterosuperior, posterior). Unfortunately, no guidance can be found in the current lexicon of the Terminologia Anatomica (the international standard for anatomic nomenclature), RadLex (RSNA radiology lexicon), or SNOMED CT (Systematized Nomenclature of Medicine–Clinical Terms).

Given the absence of formal guidance, standardization to “inferior PMF” and “superior PMF” is recommended for future publications, as has been done with recent research on the hypermobile LM [64]. Beyond honoring the original description of these fascicles, this recommended terminology is anatomically correct and simplifies competing naming conventions into a parsimonious directional system that accurately reflects the PMF positions at the popliteal hiatus and PMF attachments to the inferior and superior margins of the LM posterior horn.

Perimeniscal stabilizers can have a variable MRI appearance, and the ultimate clinical significance of various anatomic structures may need to be determined dynamically at arthroscopy. However, in order to maximize the value of routine MRI exams, (1) clinicians are encouraged to communicate specific areas of clinical concern and (2) radiologists are encouraged to use skin markers to call attention to specific areas of patient concern. For example, if the clinician suspects PMF insufficiency and a hypermobile lateral meniscus (e.g., lateral knee pain, painful squatting, locking sensation, positive figure-4 test on physical exam) [66, 67], this specific clinical concern (rather than just “knee pain”) should be communicated to the radiologist. In this clinical context (or an MRI showing an ACL tear), the radiologist is encouraged to specifically assess the status of the PMFs and evaluate for an abnormal appearance associated with the meniscocapsular junction (e.g., vertical signal abnormality, posterior pericapsular edema, widening of the popliteal hiatus, injury at the meniscofibular ligament and posterior meniscotibial ligament) [49, 64, 68]. In the absence of a clinical context, comprehensive reporting of all MRI findings may not be practical owing to the low pre-test probability of clinical significance. PMFs are not visualized in clear continuity on MRI of at least 1 in 4 asymptomatic knees [69, 70]. As a result, non-visualization of PMFs alone should not be equated with clinically symptomatic instability and does not necessarily indicate traumatic disruption. This example related to PMFs illustrates that perimeniscal stabilizer variations may represent incidental anatomic findings that do not require reporting routinely, unless these findings are thought to be relevant to the patient’s presentation or management (e.g., hypermobile LM, discoid LM, ACL tear).

##### Recommendation #4

Standardized nomenclature for the inferior and superior PMFs is recommended for future publications. For current clinical MRI reporting, however, description of PMF by location relative to the popliteal hiatus using any of the published synonyms remains acceptable.

#### Discoid meniscus

**Fig. 9:**
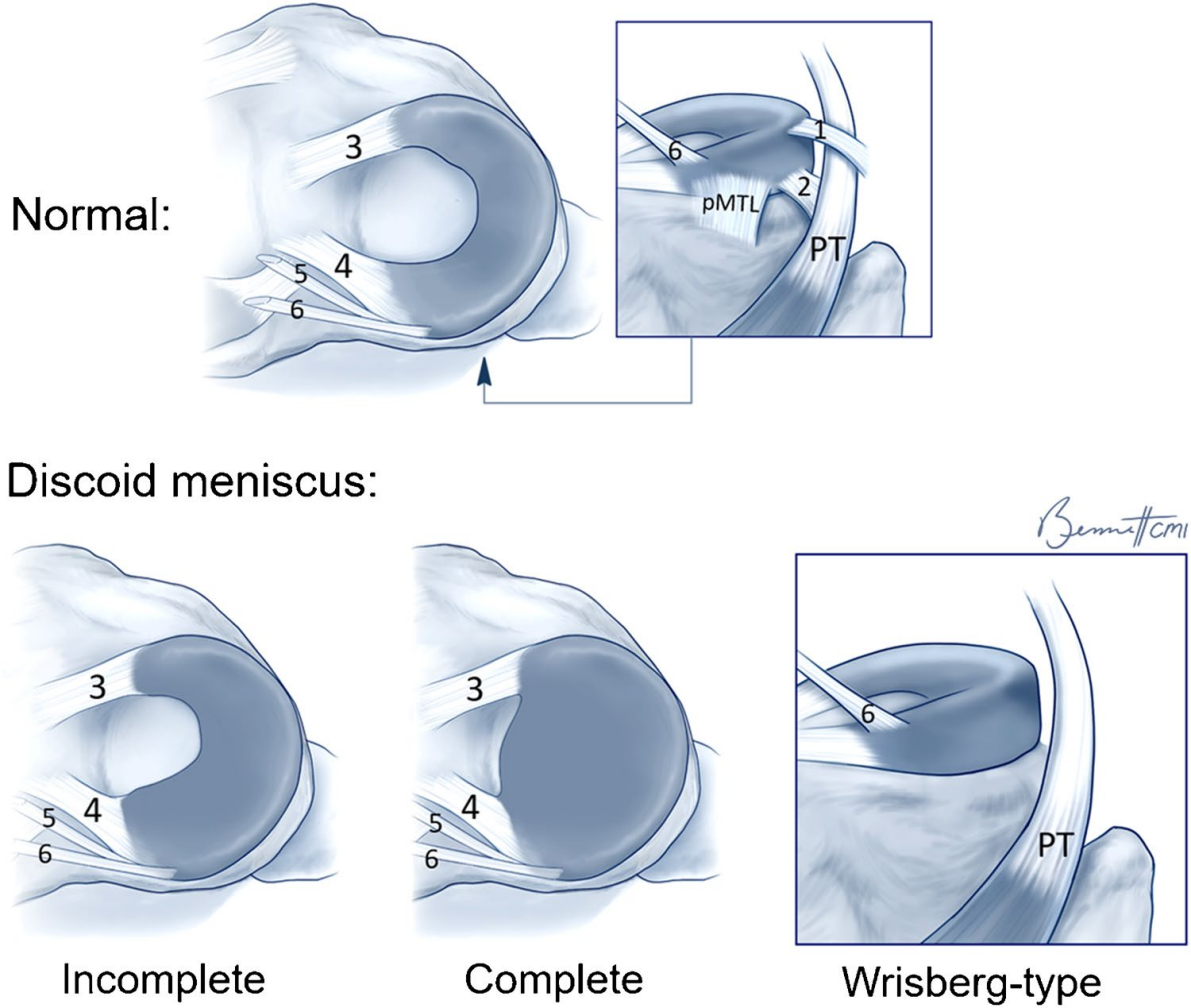
Illustrations show the normal LM and Watanabe classification of discoid LM. (1) Superior popliteomeniscal fascicle. (2) Inferior popliteomeniscal fascicle. (3) Anterior root of LM. (4) Posterior root of LM. (5) Anterior meniscofemoral ligament (Lig. of Humphry). (6) Posterior meniscofemoral ligament (Lig. of Wrisberg). PT, popliteus tendon; pMTL, posterior meniscotibial ligament. Illustration by B. Bennett, CMI @2024 The Children’s Hospital of Philadelphia. All rights reserved. Used with permission

Historically, the MRI diagnosis of a discoid LM used the criterion of 3 or more 5-mm-thick contiguous sagittal images depicting continuity (“bowties”) connecting the anterior and posterior horns [71] and focused on the Watanabe classification (type I, complete; type II, incomplete; type III, Wrisberg-type, defined by absent posterior capsular/meniscotibial attachments) (Fig. 9) [72]. Compared to the Watanabe classification, the recent Pediatric Research in Sports Medicine Society (PRiSM) classification framework categorizes a discoid LM according to four characteristics: width, height, stability, and tear pattern [73].

On MRI, description of discoid morphology appropriately includes the extent of tibial coverage on a mid-coronal image (i.e., *complete* if covering the entire lateral tibial plateau or *incomplete* if overcovering a portion of the lateral tibial plateau) and the cross-sectional shape (e.g., wedge-shaped vs. block-shaped) [15].

Ongoing research regarding the criteria for discoid LM is recognized [74, 75]. Variable diagnostic criteria are present in the literature, with published cutoffs for the LM body width of 13, 14, 15, and 17 mm [72, 76]. Another criterion mentioned in the literature is coverage of more than half the width of the lateral tibial plateau in adults [77]; this criterion is commonly used in clinical practice. Additional, similar criteria have been proposed in pediatric patients [78]. There are also less established criteria with variable thresholds (e.g., the ratio of the meniscus to the tibia, percent coverage of the meniscus [76, 79]). In addition to intrasubstance signal alteration or tear in a discoid LM, MRI reports should identify any meniscal displacement that may occur owing to insufficiency of perimeniscal stabilizers (e.g., PMFs, meniscotibial attachments).

##### Recommendation #5

Given that MRI protocols now generally use thinner (3–4 mm) slices, the “3 bowties” criterion for diagnosing a discoid LM should be abandoned. Currently, diagnosis of a discoid meniscus is appropriate on a mid-coronal slice when the width of the body segment measures 15 mm or more [72].

### Meniscal tears and associated findings

In this section, we discuss nomenclature related to intrameniscal signal alteration, tear classification systems, tear patterns, meniscal extrusion, and judicious restraint in MRI reporting.

#### Intrameniscal signal alteration and numerical grading

Historically, meniscal signal on MRI was described commonly as grade 1 (globular/punctate, not extending to an articular surface), grade 2 (linear, not extending to a meniscal surface), and grade 3 (extending to at least one meniscal surface). Numerical grading has some benefits in terms of report brevity, data mining for research (e.g., OA scoring systems such as MOAKS and WORMS), and AI tools [80].

Numerical grading systems, however, are often modified and can create confusion. Indeed, heterogeneous meniscal grading scales have been used to categorize meniscal signal (location and extent), meniscal morphology, and diagnostic confidence [81–87].

Given the proliferation of various grading systems, we endorse abandoning numerical grading for clinical MRI reporting (outside of research) in favor of describing the most salient features used to assess for a tear: surfacing signal and morphological distortion [88]. After all, a report should convey its meaning directly, without requiring the reader to look up a classification system that may or may not be specified [89]. This framework for describing meniscal signal and shape can be incorporated efficiently into structured reports with anatomical subheadings [90]. When implemented as common data elements within structured templates, this descriptive vocabulary ensures consistent terminology across readers, institutions, and time points [91].

The fundamental distinction between MRI intrasubstance signal (frequent in asymptomatic individuals) versus surfacing signal (generally representing a tear) is an issue of primary importance for clinical management and has good inter-reader agreement. However, several investigations have indicated that there is a continuum from meniscal degeneration to tear [35, 92]. Intrameniscal signal indicative of degeneration on MRI can be important as it relates to future poor outcomes (e.g., 5 times the odds of developing accelerated knee osteoarthritis) [93]. Data from the Osteoarthritis Initiative show that linear intrasubstance (grade 2) signal *not* contacting a MM surface is at substantially increased risk for developing a tear (HR 18.2; 95% CI 8.3–39.8) [94]. While most knees with linear intrameniscal signal did not progress to a tear in this study, 22% did.

On histologic examination, intrasubstance signal commonly corresponds to mucoid degeneration and/or calcification, which makes diagnosis of meniscal tears more difficult [92]. For example, chondrocalcinosis in older adults produces intrameniscal signal that can mimic or obscure meniscal tears on MRI, reducing diagnostic sensitivity to 78–89% and specificity to 71–72% [95]. These elevated false-positive and false-negative rates suggest another reason to report intrasubstance signal alteration.

Besides intrameniscal signal alteration caused by degeneration, other causes may be important to consider including intrasubstance tears, contusions, and ossicles.

- With discoid menisci, reporting the presence and location of intrameniscal signal alteration is crucial because tears may be exposed after saucerization during arthroscopy [96].
- Meniscal “contusion” as a diagnostic term should be reserved for acute, trauma-induced intrameniscal hyperintense signal that is ill-defined or amorphous (does not meet morphologic criteria for a tear). Unlike degenerative meniscal signal, trauma-related intrameniscal signal resolves in approximately 80% of patients by 2 years [97].
- Meniscal ossicles can cause altered signal intensity, most commonly at the MM posterior root region. Although reported as a vestigial (congenital) remnant in the past, these ossicles can develop in adults in association with posterior root injury (tear or avulsion) or osteoarthritis [98–100].

##### Recommendation #6

Clinical MRI reports should phase out numerical grading of meniscal signal in favor of descriptive text. Clear descriptive text emphasizing both surfacing signal and morphologic distortion criteria is recommended [88].

#### Tear classification systems

Numerous meniscal tear classification systems are published in the medical literature since 2011 (e.g., ISAKOS [101], BASK classification of meniscal lesions by treatability [102], LaPrade classification of root tears [29], Forkel classification of LM root tears [103] with subsequent modification [104], LMORT classification of LM oblique radial tears [105] with subsequent modification [106], Choi MRI classification of MM posterior root lesions [107], Asan classification of MM posterior root tears [108], radial tear classifications by Nakata [109] and Chahla [110], and MM ramp lesion classifications by Thaunat [111], Keyhani [112], Greif [113], Tollefson [114], and Tessitore [115]).

Of these classifications, the most commonly used in the literature are ISAKOS and LaPrade. Most classification systems are developed by arthroscopic surgeons. Common elements in classification systems include the tear pattern, location, and extent, which are relevant for surgical decision-making.

**Fig. 10:**
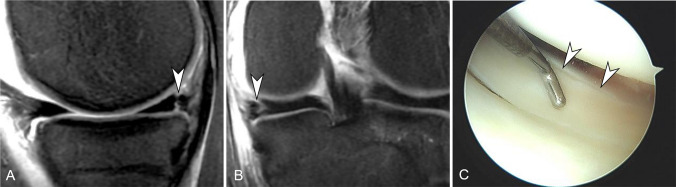
Longitudinal-vertical tear in a 15-year-old boy with ACL tear. Sagittal T2-weighted fat-suppressed MRI (**A**), coronal proton-density-weighted fat-suppressed MRI (**B**), and arthroscopic view (**C**) show complete longitudinal-vertical tear (arrowheads) of the posterior horn of the medial meniscus

**Fig. 11:**
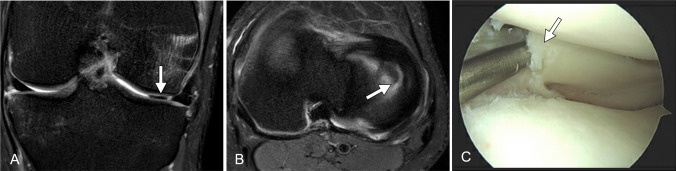
Vertical flap tear (historically termed a “parrot-beak” tear at arthroscopy) in a 25-year-old man with ACL tear. Coronal proton-density-weighted fat-suppressed MRI (**A**), axial proton-density-weighted fat-suppressed MRI (**B**), and arthroscopic view (**C**) depict a radial oblique configuration of tear involving the body and anterior horn of the lateral meniscus with a displaced meniscal flap (arrows) along the free edge

**Fig. 12:**
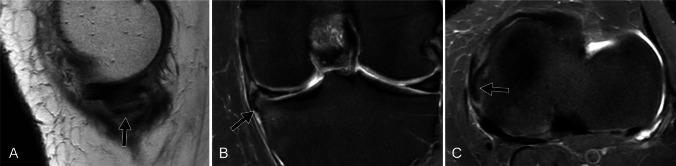
Horizontal flap tear in a 58-year-old man. Sagittal proton-density-weighted MRI (**A**), coronal proton-density-weighted fat-suppressed MRI (**B**), and axial proton-density-weighted fat-suppressed MRI (**C**) demonstrate horizontal flap tear of the body of the medial meniscus with meniscal flap (arrows) displaced into the medial meniscotibial recess. Note the bone marrow edema-like signal along the medial margin of the tibia underlying the flap, which can be a useful secondary sign when looking for a tear

The most comprehensive formal framework is the ISAKOS classification system [101]. Developed by an international expert committee for arthroscopic documentation (not MRI reporting), this system classifies tears across multiple dimensions: tear pattern (longitudinal-vertical, horizontal, radial, vertical flap, horizontal flap, complex), tear location (meniscal segment [anterior horn, body, posterior horn] and rim width [peripheral, middle, inner zones]), depth (“partial” vs. “complete”), tear length (in mm), and tissue quality (degenerative vs. nondegenerative) (Figs. 10, 11, and 12). Tear location relative to the popliteal hiatus is also included; this area lacks a capsular attachment and is flagged owing to decreased vascularity.

While the ISAKOS classification system is arguably the most detailed and widely cited classification system, it does not include important variables such as tear displacement and meniscal extrusion. A radiologist reporting only ISAKOS-defined categories could omit these critical findings. This argues for MRI reporting with a descriptive framework that incorporates ISAKOS-compatible vocabulary (e.g., pattern, location, depth, zone) rather than an unyielding template for guiding clinical decision-making. Furthermore, with over a dozen classification systems for meniscus tears so far, rigid adherence to incorporating all systems into reports is impractical.

No single meniscus tear classification system has emerged as definitively superior. Systematic reviews have found no system endorsed as most robust for MM posterior root tears [32] and limited consistency across ramp lesion classification systems [116].

Classification systems promote a shared vocabulary and efficient identification of clinically relevant findings. However, validation studies comparing MRI to arthroscopy reveal limitations. While correlation is generally good to excellent, notable discrepancies occur in assessing rim width and tissue quality. Additionally, the length of tears diagnosed by MRI tends to be larger than on arthroscopy [117].

##### Recommendation #7

**Fig. 13:**
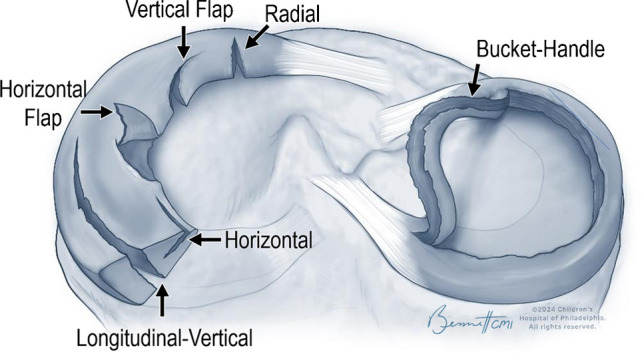
Illustration of meniscal tear classification. Illustration by B. Bennett, CMI @2024 The Children’s Hospital of Philadelphia. All rights reserved. Used with permission

Rather than rigidly adhering to a single meniscal tear classification system, we recommend using standardized descriptive terminology (e.g., horizontal, longitudinal-vertical, radial, complex) that draws from existing classification systems to provide a shared vocabulary relevant to clinical decision-making (Fig. 13).

#### Tear patterns

Important inconsistencies related to tear pattern terminology are present in classification systems and clinical practices. Terms such as “macerated,” “multidirectional,” “multidimensional,” “parrot-beak,” and “oblique” are absent from the widely accepted ISAKOS classification system, but they persist in clinical practice and contribute to inconsistent reporting. “Maceration,” for example, is variably used to refer to degenerated meniscal tissue with volume loss, fraying, and/or tearing, sometimes complex tearing. Clarity can be enhanced when tissue quality and tear pattern descriptors are reported independently. To further promote standardized reporting and alignment with ISAKOS, the legacy descriptors “multidirectional” and “multidimensional” can be replaced with “complex.”

Historically, the term “parrot-beak” tear has been used inconsistently in the literature, referring variously to an oblique tear (intermediate between radial and longitudinal-vertical orientations) or a curved radial tear with or without a displaced flap. Under the ISAKOS classification, its morphology may therefore be categorized differently depending on its appearance: as a radial tear when nondisplaced, a vertical flap tear when the displaced fragment is vertically oriented, or even a complex tear when multiple tear planes are present. In the ISAKOS framework (including the ISAKOS tear pattern diagram), the “parrot-beak” morphology corresponds to the vertical flap tear pattern. Although the term “radial oblique” may anatomically describe the morphology of this tear more precisely (i.e., a radial tear with a curved or oblique course), in practice radiologists often abbreviate this to “oblique,” grouping it together with other obliquely oriented tears that lack a radial component and thus creating ambiguity. We therefore recommend using the term “vertical flap” for greater consistency with ISAKOS terminology (Fig. 11).

**Fig. 14:**
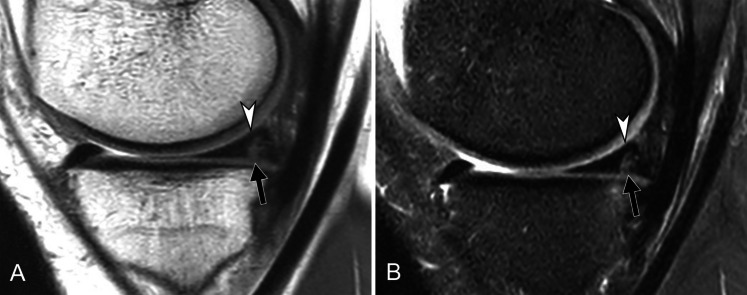
Sagittal proton-density-weighted MRI (**A**) and T2-weighted fat-suppressed MRI (**B**) of a 27-year-old man with ACL tear demonstrate partial-thickness longitudinal-vertical tear of the posterior horn of the medial meniscus (arrows) sparing the superior surface of the meniscus (arrowheads)

Inconsistency and ambiguity in the use of the terms “complete,” “incomplete,” and “partial” also are present. These terms conflate three independent dimensions: thickness (full- vs. partial-thickness with respect to the superior and inferior meniscal surfaces), radial extent (whether a tear fully transects the meniscus from free edge to periphery), and circumferential extent (the length of a longitudinal-vertical tear) (Fig. 14).

Commonly used to describe radial and horizontal tears, the terms “complete,” “incomplete,” and “partial” generally refer to the width of meniscal involvement (from the free edge to periphery), but these descriptors can become problematic when the dimension is not specified.

**Fig. 15:**
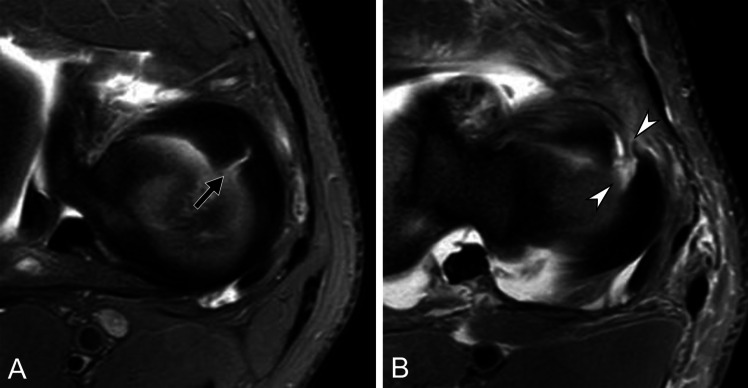
Partial versus complete radial tears. Radial tears of the anterior horn and body junction of the lateral meniscus in two different patents with ACL rupture depicted on axial proton-density-weighted fat-suppressed MRI (**A**, **B**). A 27-year-old man with incomplete, or partial-width, radial tear of the lateral meniscus (arrow, **A**) that spares the peripheral third of the meniscus. A 17-year-old man with complete, or full-width, radial tear of the lateral meniscus extending from free edge to periphery (arrowheads, **B**) with 5 mm gap

- For radial tears, "complete" (or "full-width") indicates extension from the free edge to the capsular margin, while "partial" (or "partial-width") indicates the tear extends only partway across the meniscal width. Surgeons are often interested in the radial extent of the tear (reported in mm or as an estimated percentage of the meniscal width) (Fig. 15).
- Similarly, horizontal tears are described as “complete” (or “full-width”) when they extend from the free edge to the periphery of the meniscus, while “incomplete” (or “partial-width”) tears involve less than the entire width of the meniscus.

To minimize ambiguity, MRI reports and publications using the terms “complete,” “incomplete,” or “partial” should ideally specify the tear pattern with the intended dimension (e.g., depth, radial extent, or circumferential extent) [5].

**Fig. 16:**
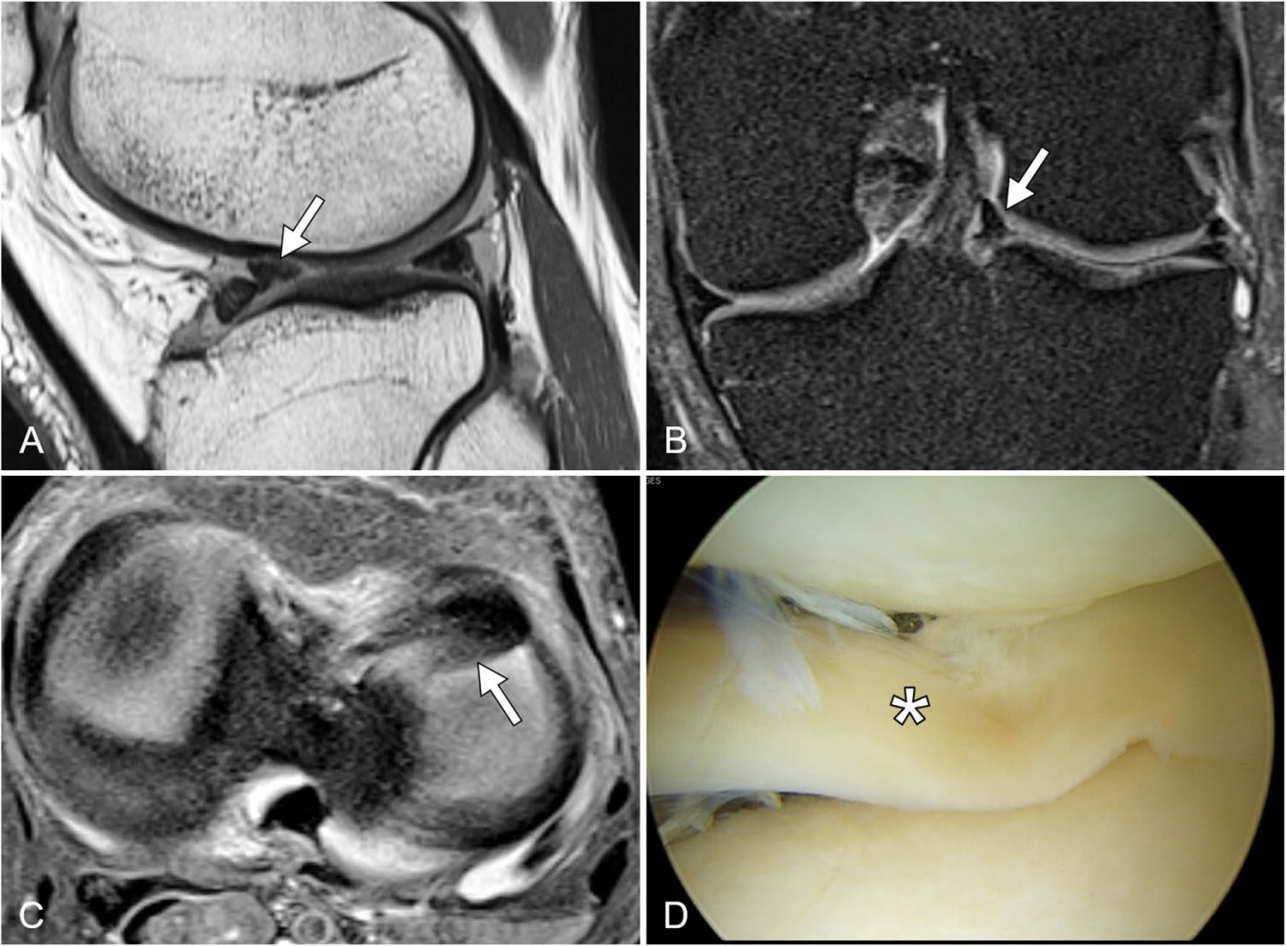
Bucket-handle tear of the lateral meniscus in a 36-year-old man. Sagittal proton-density-weighted MRI (**A**), coronal proton-density-weighted fat-suppressed MRI (**B**), and axial proton-density-weighted fat-suppressed MRI (**C**) show central fragment (arrows) displaced into the intercondylar notch. Arthroscopic view (**D**) demonstrates large lateral meniscus fragment (*) displaced anteriorly and medially located in the intercondylar notch

- Longitudinal-vertical tear depth involving both the superior and inferior surfaces may be described most simply as “full-thickness.” In the ISAKOS system, “complete” is used to describe these tears (i.e., tear depth), but “complete” is also used to describe longitudinal-vertical tears involving the complete circumferential extent of the meniscus from anterior to posterior horn. To disambiguate the usage of the term “complete,” rather than describing a “complete longitudinal-vertical tear,” we favor describing a bucket-handle tear that extends from the anterior horn to the posterior horn (Fig. 16).

##### Recommendation #8

**Fig. 17:**
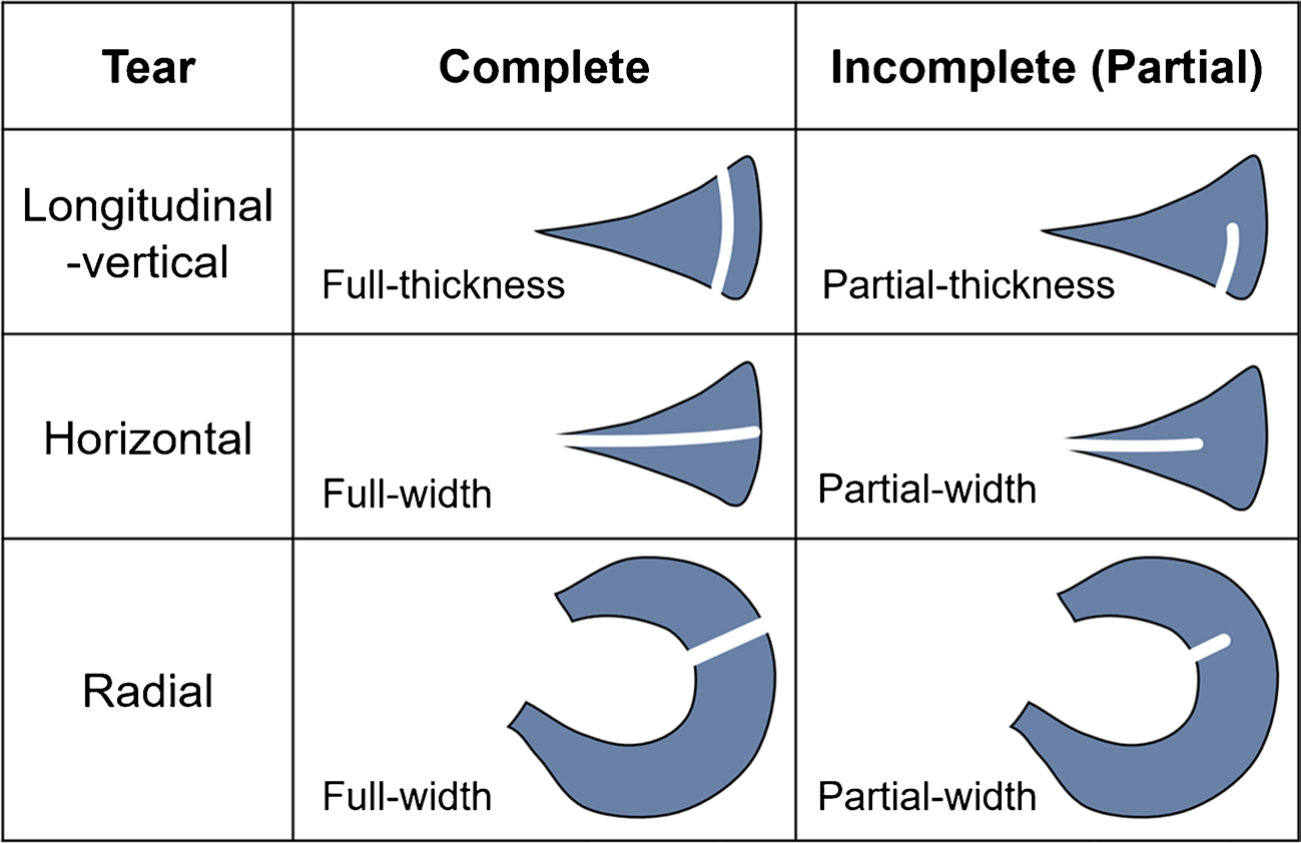
Complete vs incomplete (or partial) tears. To minimize ambiguity, the terms “complete” and “incomplete” (or “partial”) should specify the tear pattern and tear extent

When reporting a meniscus tear, the descriptors “complete”, “incomplete”, or “partial” can be clarified by also specifying the tear pattern and extent (Fig. 17).

#### Meniscal extrusion

**Fig. 18:**
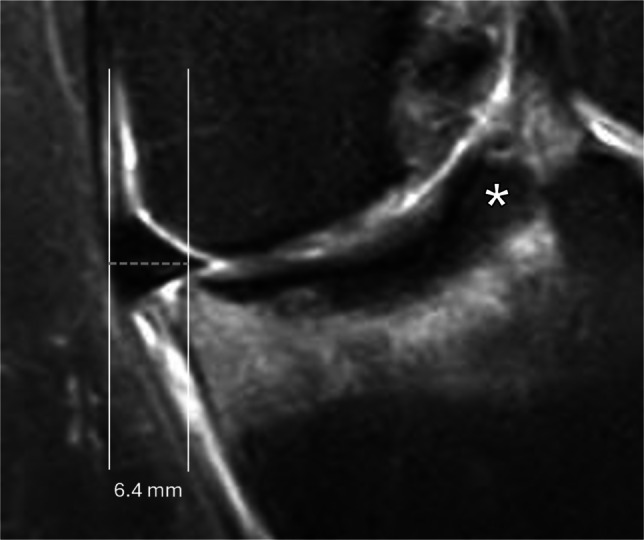
Pathological extrusion on the medial meniscus in a 69-year-old woman with complete radial tear of the posterior root of the medial meniscus and subchondral insufficiency fracture of the medial tibial plateau. Coronal proton-density-weighted fat-suppressed MRI depicts measurement of extrusion performed on the image with the greatest volume of the medial tibial spine (*), as a distance between peripheral chondroosseous margin of the tibia and outer edge of the meniscus

Meniscal extrusion is defined as radial displacement of the meniscus beyond the outer margin of the tibial plateau, most commonly measured on coronal MRI through the meniscal body. Extrusion is generally measured as the perpendicular distance from the outer edge of the tibial plateau to the outermost margin of the meniscal body on a coronal image (Fig. 18). However, substantial heterogeneity exists in how the reference coronal slice is selected. The Meniscus International Network Study Group has recommended that extrusion be measured at the mid-tibial plateau level (reported in mm) [118]. A meniscal extrusion index has also been proposed to account for individual variations in patient size [119].

For the MM, a conventional threshold for abnormal extrusion is > 3 mm (range, 2.5–4 mm) [120]. Given the ongoing debate over thresholds (2.5 mm vs. 3 mm vs. 4 mm), reporting the actual measurement (in mm) is more informative than a binary designation of “present” or “absent.”

For the LM, > 2 mm has been proposed as a cutoff point for abnormal extrusion, but the absence of extrusion should not be used to exclude a LM posterior root tear [120]. Compared to the MM, extrusion of the LM is much less common because the meniscofemoral ligaments and PMFs can restrain LM displacement [121–124].

Extrusion may be secondary to a root tear, radial tear, medial meniscotibial ligament injury, or osteoarthritis. Owing to biomechanical overloading, extrusion can result in bone marrow edema (stress reaction), subchondral insufficiency fractures, and accelerated osteoarthritis [125, 126]. When available, alignment can be reported with extrusion findings. Varus malalignment is independently associated with medial extrusion (OR 1.3; 95% CI 1.1–1.7), and valgus malalignment with lateral extrusion (OR 2.2; 95% CI 1.5–3.2) [127].

Extrusion should be differentiated from a meniscal tear with a flap of tissue displaced into the meniscotibial or meniscofemoral recess. Although MRI differentiation between these two entities can be challenging, they have fundamentally different pathophysiology and management implications [128]. With extrusion, the entire meniscus body segment is shifted relative to the tibial plateau and commonly retains its triangular shape. In contrast, with a typical displaced flap from a tear in the meniscal body segment, the parent meniscus shows findings of a tear with deformity (e.g., truncation, donor-site defect). Associated secondary signs also differ. The subchondral bone marrow edema seen with extrusion tends to be more centrally located, while the reactive bone edema with a displaced flap at the medial meniscotibial recess is located characteristically at the medial rim of the tibial plateau [129] (Fig. 12). Of note, both meniscal extrusion and a displaced flap may coexist.

##### Recommendation #9

Extrusion should be reported using standardized terminology that includes the numeric measurement in millimeters, the compartment (medial or lateral), and the coronal reference slice (preferably at the mid-tibial plateau level), rather than relying on variable binary thresholds that differ across the literature.

#### Restraint in reporting

The radiologist’s role in meniscal tear reporting is to provide an accurate description—not to infer tear attributes such as duration, etiology, treatment, or clinical significance.

Meniscal tear duration (“acute” vs. “chronic”) often cannot be definitively established by MRI without a prior comparison exam. While horizontal tears in older patients with associated chondrosis may suggest a degenerative process, and longitudinal-vertical tears in young patients following an acute injury may suggest a traumatic mechanism, many tears occur at the intersection of degenerative predisposition and mechanical loading. Additionally, MRI cannot determine whether a pre-existing “degenerative tear” has become newly symptomatic following a minor traumatic event (a common clinical scenario). Experts emphasize that a tear can occur in either a degenerative or non-degenerative meniscus and that degenerative meniscal lesions may be incidental findings on knee MRI in older individuals [102, 130].

Etiological labeling of a tear (“traumatic” vs. “degenerative”) can have consequences that extend beyond communication to the clinical physician. Designating a tear as “traumatic” may carry medicolegal implications in the context of workers’ compensation, personal injury, or disability claims. Designating a tear as “degenerative” may trigger insurance denial of coverage for arthroscopic debridement or repair, potentially limiting patient access to appropriate care. Unless the clinical context is unambiguous, the MRI report should describe morphology, location, and associated findings without asserting an etiology or acuity.

MRI reports should avoid terms that suggest surgical appropriateness (e.g., “repairable,” “unrepairable”). MRI is generally regarded as a poor predictor of meniscal tear reparability [131–133], although the literature is not entirely uniform [134]. In general, the terms “stable” and “unstable” should also be avoided when characterizing a tear in an MRI report. Reparability is ultimately determined intraoperatively based on factors that cannot be definitively established on MRI (e.g., vascularity, tissue quality, and instability during mechanical probing) [47, 135]. Moreover, tear types that were considered unrepairable a decade ago (e.g., radial tears, root tears, tears in avascular zones) now may be considered for repair in selected circumstances [136, 137]. In addition to the knee MRI findings, of course, treatment decisions are influenced by multiple other factors, including patient age, symptoms (e.g., pain level, mechanical symptoms), and comorbidities (e.g., BMI, lower extremity malalignment).

**Fig. 19:**
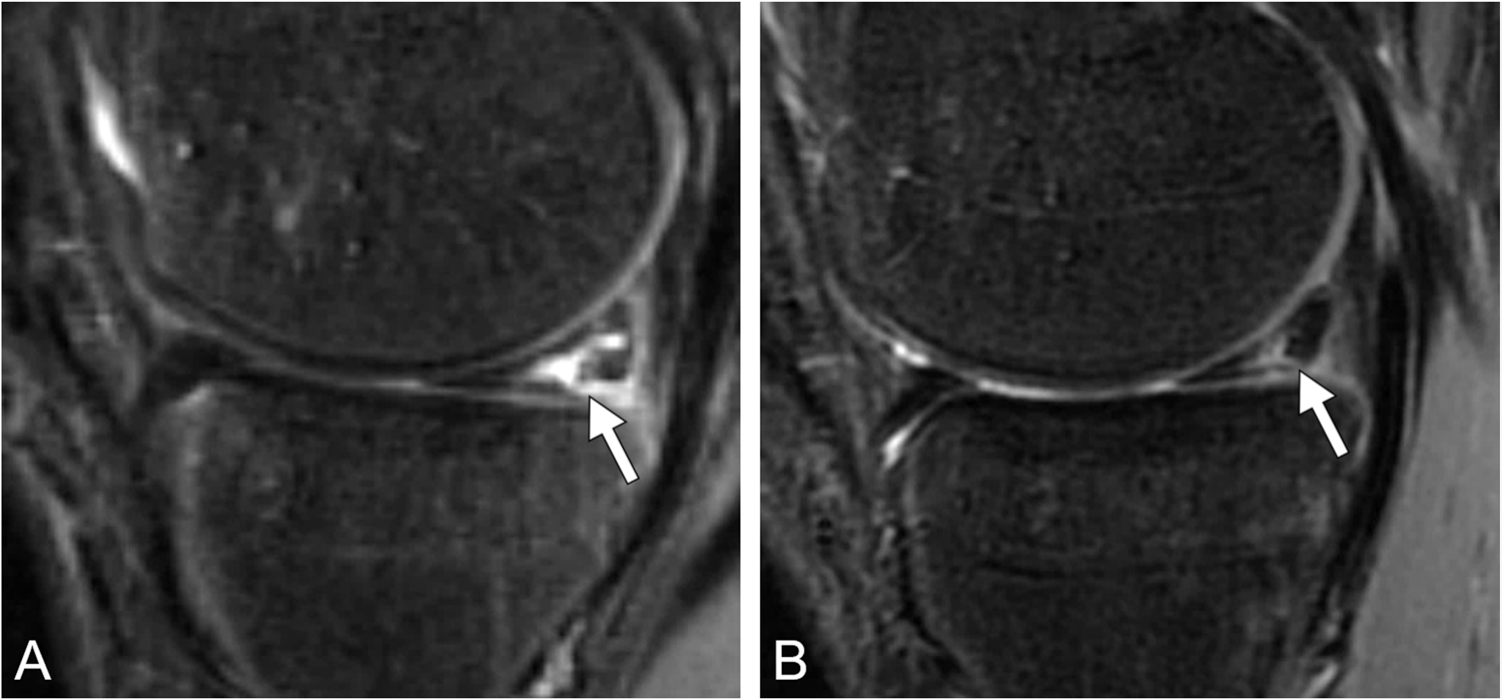
Postoperative meniscus. Preoperative sagittal T2-weighted fat-suppressed MRI (**A**) of a 22-year-old woman with chronic knee pain demonstrates branching linear fluid signal intensity in the medial meniscus posterior horn (arrow) diagnostic of a complex tear. Sagittal T2-weighted fat-suppressed MRI (**B**) obtained one year and one month after medial meniscus repair depicts intermediate signal intensity reparative tissue at site of surgery (arrow)

**Fig. 20:**
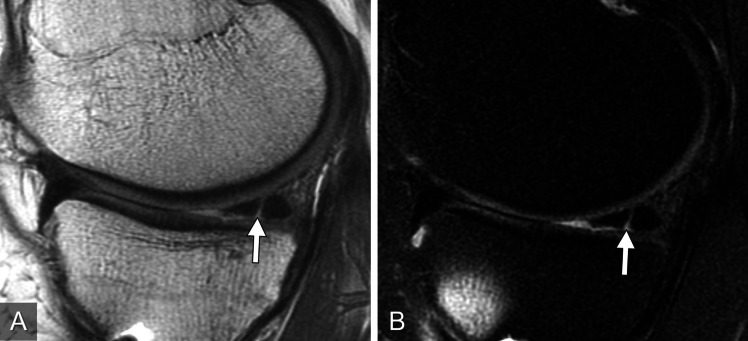
Recurrent tear of the medial meniscus in a 30-year-old woman with history of ACL reconstruction who underwent repair of a bucket-handle tear of the medial meniscus. Sagittal proton-density-weighted MRI (**A**) and sagittal T2-weighted fat-suppressed MRI (**B**) obtained one year after meniscus repair demonstrate linear fluid-like signal (arrows) surfacing at the medial meniscus posterior horn indicative of a tear, which was confirmed arthroscopically and treated with partial meniscectomy

Restraint in knee MRI reporting may also be appropriate for the postoperative meniscus. After partial meniscectomy or repair, the usual findings of a tear may be present in an untorn meniscus (e.g., blunting deformity or intermediate signal contacting the meniscal surface). The most reliable criterion for recurrent tear at the site of prior meniscal surgery is high T2-weighted signal extending to the meniscus surface, a displaced fragment (PPV 100%), or a change in signal pattern compared with baseline MRI (PPV 99.4%) [138]. The term “residual tear” generally should be avoided as it implies failure of surgical treatment. Instead, descriptive language regarding meniscal morphology (e.g., truncation, volume loss, cleft) or a signal abnormality at the meniscectomy margin may be appropriate if postoperative change versus recurrent tear is unclear (Figs. 19 and 20). Importantly, MRI interpretation of the postoperative meniscus should be performed in conjunction with preoperative imaging and the operative report whenever possible [6, 139–141].

##### Recommendation #10

MRI reports should describe meniscal tear morphology, location, and associated findings without inferring etiology (“traumatic” vs. “degenerative”), chronicity (“acute” vs. “chronic”), or surgical suitability (“repairable,” “unrepairable,” “stable,” “unstable”). For the postoperative meniscus, the term “residual tear” should generally be avoided in favor of descriptive language that distinguishes expected postoperative change from findings suspicious for recurrent tear.

This work is subject to limitations inherent to consensus methodology, including potential expert bias and the absence of prospective validation data. The recommendations reflect current best evidence and prevailing expert opinion but may require revision as new data emerge, such as with root zone definitions and extrusion thresholds, which remain active areas of investigation. Additionally, the practical impact of adopting these recommendations on inter-reader agreement, clinical decision-making, and patient outcomes has not yet been assessed. Finally, lexical inconsistencies remain in the field, such as the interchangeable use of noun modifiers (e.g., meniscus tear) and adjectival forms (e.g., meniscal tear); standardization of terminology across societies may warrant future attention.

In conclusion, this interdisciplinary consensus identifies key areas of nomenclature variability relevant to both meniscal anatomy and tear characterization. Across both domains, a consistent principle emerges. Descriptive anatomic language—specifying what is seen and where—can be more reproducible and clinically actionable than reporting variable numerical grades and evolving classifications. We offer ten pragmatic recommendations related to common issues in MRI interpretation: meniscal roots, vascularity zones, perimeniscal stabilizer anatomy and nomenclature, discoid meniscus morphology, intrameniscal signal grading, tear classification frameworks, specific tear pattern descriptors, extrusion measurement, and reporting boundaries. Adoption of these recommendations has the potential to reduce miscommunication between radiologists and surgeons, improve consistency across institutions, and support more reliable data for clinical research. As meniscal preservation strategies continue to evolve, standardized reporting will be essential to ensure that imaging communications match the precision of evolving surgical techniques. Future work should prospectively assess the impact of these recommendations on inter-reader agreement, clinical decision-making, and patient outcomes.

## Data Availability

Not applicable.

## Abbreviations

CI: Confidence interval
HR: Hazard ratio
ISAKOS: International Society of Arthroscopy, Knee Surgery and Orthopaedic Sports Medicine
LM: Lateral meniscus
MCL: Medial collateral ligament
MM: Medial meniscus
OR: Odds ratio
PMF: Popliteomeniscal fascicle
PRiSM: Pediatric Research in Sports Medicine Society
